# The diagnostic power of CD117, CD13, CD56, CD64, and MPO in rapid screening acute promyelocytic leukemia

**DOI:** 10.1186/s13104-020-05235-7

**Published:** 2020-08-26

**Authors:** Vinh Thanh Tran, Thang Thanh Phan, Hong-Phuoc Mac, Tung Thanh Tran, Toan Trong Ho, Suong Phuoc Pho, Van-Anh Ngoc Nguyen, Truc-My Vo, Hue Thi Nguyen, Thao Thi Le, Tin Huu Vo, Son Truong Nguyen

**Affiliations:** 1 The Laboratory D Unit, Clinical Cancer Center, Cho Ray Hospital, 201B Nguyen Chi Thanh Street, Dist. 5, Ho Chi Minh City, 700000 Vietnam; 2 Biomolecular & Genetic Unit, Clinical Cancer Center, Cho Ray Hospital, Ho Chi Minh City, 700000 Vietnam; 3 Faculty of Biology and Biotechnology, University of Science, VNU-HCM, Ho Chi Minh City, 700000 Vietnam; 4 Department of the Vice-Minister, Ministry of Health, Hanoi, 100000 Vietnam

**Keywords:** CD117, CD13, CD56, CD64, MPO, PML–RARA, APL

## Abstract

**Objective:**

The same immuno-phenotype between HLA-DR-negative acute myeloid leukemia (AML) and acute promyelocytic leukemia (APL) causes APL rapid screening to become difficult. This study aimed to identify the associated antigens for APL and the best model in clinical uses.

**Results:**

A total of 36 APL (*PML–RARA*+) and 29 HLA-DR-negative non-APL patients enrolled in this study. When a cut-off point of 20% events was applied to define positive or negative status, APL and non-APL patients share a similar immuno-phenotype of CD117, CD34, CD11b, CD13, CD33, and MPO (*P* > 0.05). However, expression intensity of CD117 (*P* = 0.002), CD13 (*P* < 0.001), CD35 (*P* < 0.001), CD64 (*P* < 0.001), and MPO (*P* < 0.001) in APL are significantly higher while CD56 (*P* = 0.049) is lower than in non-APL subjects. The Bayesian Model Averaging (BMA) analysis identified CD117 (≥ 49% events), CD13 (≥ 88% events), CD56 (≤ 25% events), CD64 (≥ 42% events), and MPO (≥ 97% events) antigens as an optimal model for APL diagnosis. A combination of these factors resulted in an area under curve (AUC) value of 0.98 together with 91.7% sensitivity and 93.1% specificity, which is better than individual markers (AUC were 0.76, 0.84, 0.65, 0.82, and 0.85, respectively) (*P* = 0.001).

## Introduction

APL is a hematological malignancy that is characterized by a translocation between chromosome 15 and chromosome 17, the t(15;17)(q22;q11) translocation. It leads to the formation of the Promyelocytic leukemia–Retinoic acid receptor alpha (*PML–RARA*) fusion gene in hematopoietic stem cells [1–3]. This fusion can be detected in > 95% APL patients with three major transcript subtypes (bcr1, bcr2, and bcr3) depend on the breakpoints of the *PML* gene, and some rare subtypes [3].

Regarding the *PML–RARA* fusions, all-*trans* retinoic acid (ATRA) and arsenic trioxide are highly effective agents that are combined in a current treatment method for APL patients [4–7]. According to the recommendations of European LeukemiaNet, treatment with ATRA should be immediately-initiated to prevent the risk of severe bleeding, whereas a rapid confirmation of *PML–RARA* fusions is mandatory in all cases [8]. This diagnostic test has been recommended to perform on the bone marrow-cells, by the fluorescence in situ hybridization (FISH) and real-time quantitative polymerase chain reaction (RQ-PCR) methods [8]. Of which, PCR was used as the gold-standard method for over ten years [9]. Besides, the immunostaining with anti-PML antibodies can be used to surrogate for genetic testing. However, this method requires an experienced examiner to do while results are less reproducible [8].

Some immunophenotypic markers as CD34, CD117, HLA-DR, CD13, CD9, CD18, CD2, and CD11a, CD11b might be helpful to guide the APL diagnosis in a fasting method with turnaround time just in two hours [10]. Previous studies have shown that combination some these antigens help to detect APL with high accuracy, sensitivity, and specificity [11–16]. In reality, the morphology and immuno-phenotype of APL are different from HLA-DR-positive AML. Whereas, an analogous immuno-phenotype can be found in certain-cases of HLA-DR-negative AML [17–19]. This phenomenon causes APL screening to become much more difficult in clinically. All of the above studies investigated the diagnostic values of flow cytometric antigens that used a control group containing > 50% HLA-DR-positive AML patients [11–16]. A few reports mentioned the role of these markers in comparison with a similar phenotype control group [20, 21]. We compared the antigen expression level between APL and HLA-DR-negative non-APL patients and identified the associated markers with APL together with an optimal model in clinical diagnostics.

## Main text

### Materials and methods

#### Patients

A total of 65 newly diagnosed AML patients with HLA-DR-negative enrolled in this study at Cho Ray hospital from Feb-2016 to March-2020 (approval number 602-BVCR-HDDD) (Additional file 1: Figure S1). Because of a retrospective study, patients were not requested to write consent forms. Among them, 36 cases were confirmed APL by the presence of t(15:17) translocation (median 72.1% investigated myeloid cells) and *PML–RARA* fusions (median 82.9% total transcripts) (Additional file 2: Table S1). Twenty-nine remaining cases with *PML–RARA* negative results were classified into the non-APL group. APL patients presented at a median age of 46 years old, and with the white blood cell number of 8.7 × 10^9^/l, which were lower than in non-APL patients.

#### Flow cytometric analysis

In the flow cytometric analysis, a procedure with antibody-panel was performed according to the recommendations of EuroFlow [22]. Briefly, 100 µl bone marrow cells were incubated with a cocktail of antibodies for 15 min, and then with 500 µl FACS lysing solution (BD Biosciences). Centrifugation at 3000 rpm for 3 min was applied to remove supernatant and debris. Afterward, the samples were washed with 2 ml phosphate-buffered saline solution and re-suspended in 500 µl Sheat solution before acquiring on the 8-colors FACSCanto-II system (BD Biosciences, San Jose CA, USA). A percentage of positive myeloid cells (Mye.C) with each antigen was reported as in Additional file 3: Figure S2. AML with HLA-DR-negative was distinguished according to the classification criteria of EuroFlow and European LeukemiaNet [22, 23].

#### Molecular and cytogenetic analyses

The t(15;17) translocation was detected in the bone marrow cells by the FISH technique using Vysis LSI PML/RARA Dual Color, Dual Fusion Translocation Probe kit (Cat No. 01N36-020, Abbott Molecular, Illinois, USA) according to the manufacturer’s instructions. Briefly, the mono-nucleated cells were collected and treated with KCl 0.075 M solution at 37 °C/40 min and Carnoy’s fixative solution (Abbott Molecular) for 20 min. After that, cells were dropped on a positively charged slide and incubated with a 10 µl probe mixture at 75 °C/3 min and 37 °C/16–20 h. Finally, the slide was washed with SSC-NP-40 solution and stained with DAPI-II solution before analyzing by the BioView system (Abbott Molecular). The translocation signals were reviewed and calculated in ≥ 400 cells (Additional file 4: Figure S3).

For the *PML–RARA* transcripts detection, total RNA was extracted from bone marrow cells by using the QIAamp RNA Blood Mini kit (Cat No. 52304, Qiagen, Hilden, Germany). The *PML–RARA* transcripts (bcr1, bcr2, and bcr3) were detected by the RQ-PCR technique using Ipsogen PML–RARA kits (Cat No. 672123, 672213, and 672313, Qiagen, Hilden, Germany) according to the manufacturer’s instructions. PCR reactions were performed and analyzed by the RotorGene Q 5Plex HRM platform (Qiagen, Hilden, Germany). Transcript results were reported as normalized to control gene (*ABL*) copy number (Additional file 4: Figure S3).

#### Statistical analysis

The Chi-square or Fisher’s exact (frequency < 5) tests were used to compare the frequencies, while the Kruskal–Wallis rank test was used to compare the expression level of each antigen between groups. The BMA statistic was used to identify the associated markers with APL and optimal model in diagnostics. The logistic regression was used to construct the receiver operating characteristic (ROC) curve and define the cut-off point together with sensitivity, specificity, and the value under the ROC curve (area under the curve: AUC) of each antigen and optimal model in diagnosis APL. All data analyses were done by R statistical software v.3.5.1 (R foundation, 1020 Vienna, Austria). *P* < 0.05 was considered statistically significant.

### Results

#### Antigen expression between groups

**Fig. 1:**
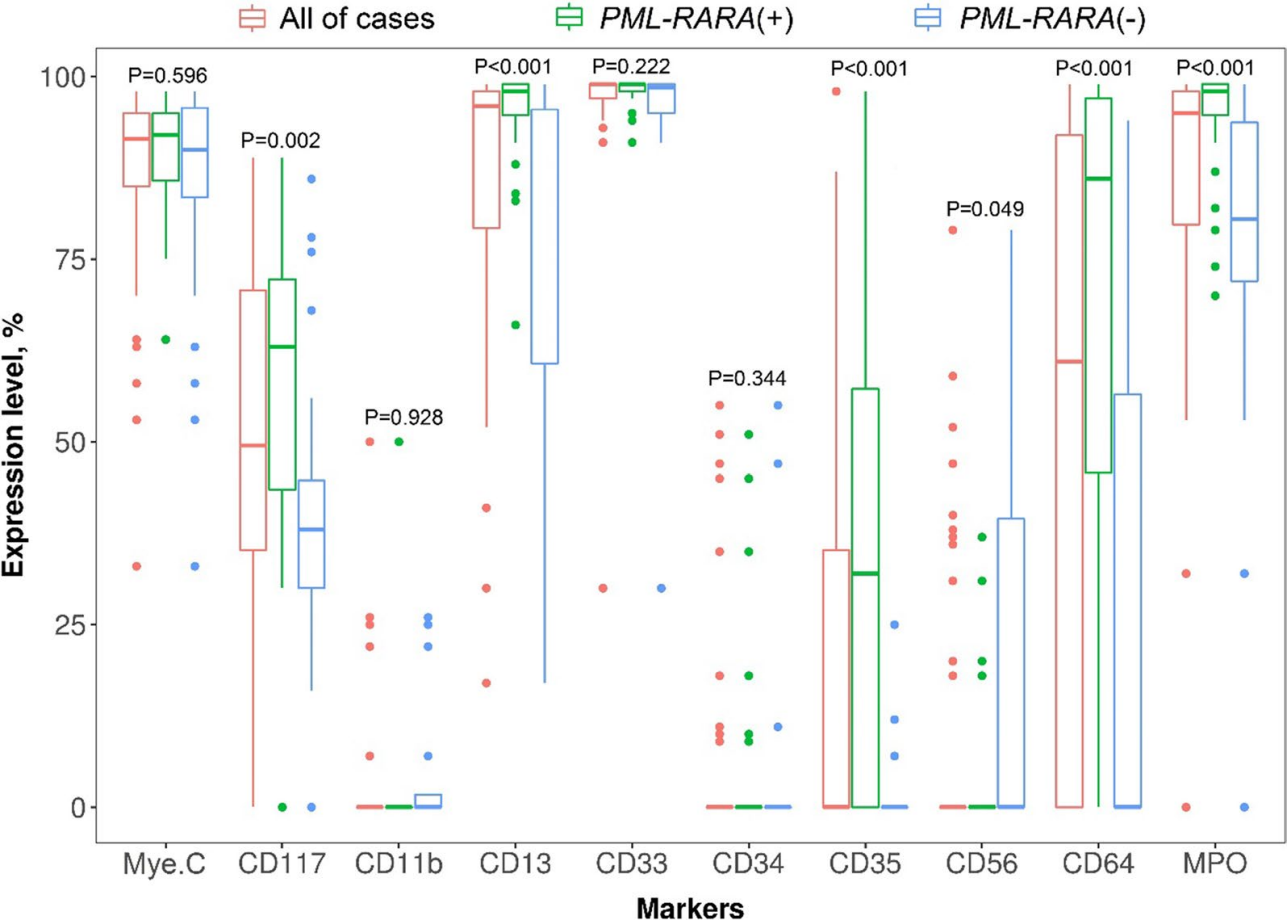
Antigen expression level between groups of *PML–RARA* status

All of 65 cases were negative with HLA-DR antigen, lymphocyte lineage (CD10, cyCD3, cyCD79a, TdT, CD3, CD5, CD7, CD8, CD19, CD20, CD22) and other markers (CD71, CD105, CD16, CD36, IREM2). Among them, data of CD11b and CD35 antigens are available only in 35 and 36 cases, respectively (Additional file 5: Table S2). When a cut-off value of 20% events was applied to define positive or negative status, most of the patients are negative with CD34 (90.8%), CD11b (88.6%), CD14 (98.5%), and CD56 (78.5%) while positive with CD117 (95.4%), CD13 (98.5%), CD33 (100%), CD64 (72.3%), and MPO (myeloperoxidase, 95.4%) (Additional file 5: Table S2). We also noted that APL patients share a similar immuno-phenotype of CD117 (*P* = 0.418), CD34 (*P* = 0.445), CD11b (*P* = 0.238), CD13 (*P* = 0.446), CD14 (*P* = 0.554), CD33 (positive in 100% cases), and MPO (*P* = 0.084) with non-APL patients. However, when the expression intensity of antigens was shown, we found that median level of CD117 (*P* = 0.002), CD13 (*P* < 0.001), CD35 (*P* < 0.001), CD64 (*P* < 0.001), and MPO (*P* < 0.001) in APL patients are significantly higher than in non-APL subjects (Fig. 1). Contrariwise, the CD56 expression level in APL patients is lower than in others (*P* = 0.049).

#### Associated factors with APL and optimal model in diagnostics

The BMA analysis has identified five factors, including CD117, CD13, CD56, CD64, and MPO, which are closely associated with APL (Additional file 6: Figure S4). The probability that these antigens linked to APL were 77%, 100%, 59%, 81%, and 96%, respectively. These factors are also included in the optimal model for APL diagnosis by the BMA analysis. Because of insufficient data, CD11b and CD35 were not included in this analysis.

**Fig. 2:**
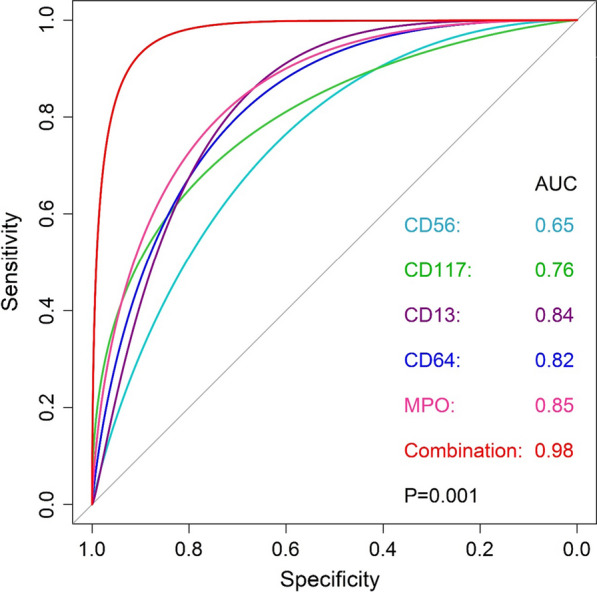
The diagnostic power of CD117, CD13, CD56, CD64, and MPO in combination

In the univariable logistic regression, APL was differentiated from non-APL with an accuracy of 76% by CD117 (AUC = 0.76; cut-off: ≥ 49% cells), 84% by CD13 (AUC = 0.84; cut-off: ≥ 88% cells), 65% by CD56 (AUC = 0.65; cut-off: ≤ 25% cells), 82% by CD64 (AUC = 0.82; cut-off: ≥ 42% cells), and 85% by MPO (AUC = 0.85; cut-off: ≥ 97% cells) (Table 1). The multivariable analysis showed that the combination of these factors resulted in a significantly increased accuracy value (AUC = 0.98, 95% CI 0.95–1.00, *P* = 0.001) (Fig. 2). The sensitivity and specificity of the optimal model in diagnosis APL were 91.7% (95% CI 80.6–100.0) and 93.1% (95% CI 82.8–100.0), respectively.

**Table 1:** Diagnostic values of each marker for the APL

Antigen	Cut-off (%)	AUC (95% CI)	Sensitivity, % (95% CI)	Specificity, % (95% CI)
CD117	≥ 49	0.76 (64.0–88.0)	72.2 (56.3–87.5)	75.9 (59.1–90.9)
CD13	≥ 88	0.84 (0.73–0.95)	90.6 (81.2–100.0)	68.2 (50.0–86.4)
CD56	≤ 25	0.65 (0.54–0.76)	41.4 (23.5–61.1)	91.7 (77.5–98.3)
CD64	≥ 42	0.82 (71.3–93.5)	84.4 (71.9–96.9)	72.7 (54.6–90.9)
MPO	≥ 97	0.85 (0.75–0.94)	63.9 (50.0–81.3)	89.7 (77.3–100.0)

### Discussion

Flow cytometry is an essential method that is used widely in the classification of AML and other hematological diseases. An advantage of this method is to give results within 2 h, that is suitable to use in fast screening of APL to minimize the risk of death caused by the disease. This approach based on the distinct profile of cell antigens between APL and other types of AML [24–27]. Most of the myeloblasts in AML express CD34, HLA-DR, CD117, CD13, CD33, and MPO antigens. Whereas, CD34, HLA-DR, and CD11b antigens are rarely-expressed by promyelocytes and myelocytes in APL [24–26]. Thus, the absence of these antigens in AML cells leading to a similar pattern to APL cells that makes difficulties in APL diagnosis. Previous studies have shown the high diagnostic values of cell antigens for APL but with a comparison to an AML control group containing a high percentage of HLA-DR-positive subjects [11–16]. Only two studies assessed the diagnostic role of cell antigens for APL in comparison to an HLA-DR-negative AML control group and showed high sensitivity, specificity, and accuracy values (98–100%) [20, 21].

In this study, we used a cut-off point of 20% events to define expression status as in previous studies [20, 21] but, no significant differences of antigen profile between APL and HLA-DR-negative AML was found (except CD56 and CD64, Additional file 5: Table S2). These results indicate that the immuno-phenotype of the non-APL and APL cases are highly closed. In the studies of Liu and Mosleh, although HLA-DR is negative in all control subjects, the expression of other antigens as CD117, CD34, CD11b, CD13, CD33, CD64, and MPO are significantly different between APL and non-APL patients [20, 21]. So, despite the high diagnostic values presented by Liu and Mosleh, a cut-off value of 20% events applied for all cell antigens might not be useful in APL differential diagnosis, at least from those with APL-like immuno-phenotype as in this study.

We assessed expression data of each marker as a continuous variable and note that the expression intensity of cell antigens (CD117, CD13, CD35, CD56, CD64, and MPO) are significantly different between APL and non-APL subjects (Fig. 1). Importantly, these antigens are significant in classifying APL, while cut-off points are optimized rather than a fixed value of 20% events (Table 1). These are different from previous studies that used HLA-DR-negative AML as the control group [20, 21]. Whereas in comparing the diagnostic performance, we noted that the combination of five markers, including CD117, CD13, CD56, CD64, and MPO, resulted in excellent accuracy (Fig. 2), which are comparable with reports of Liu and Mosleh [20, 21].

By the BMA statistics, we also noted that CD56 contributes significantly to APL screening (Fig. 2), which was just mentioned as a low expression marker in the disease compared to other types of AML [11, 13, 14, 16–20]. Clinically, patients without CD56 expression have a better prognosis compared to others when treated with the ATRA agent [27–32]. Based on this benefit of prognostics and the diagnostic power of the model (Fig. 2) together with rapidity and cost-effectiveness of flow cytometry, we suggest using this method first to identify APL and prevent risks of related complications. Also, practicians should keep in mind that a multi-colors device and an optimized panel of cell antigens can help to accelerate the prompt diagnosis. After that, confirmation of *PML–RARA* fusions by FISH and RQ-PCR techniques need to be done, according to the current recommendations [8].

### Conclusion

The results of this study indicated that the expression intensity of CD117, CD13, CD56, CD64, and MPO antigens in APL are significantly different from HLA-DR-negative AML. Besides, an optimal model combining these five markers might help to differentiate APL from APL-like immuno-phenotype AML with high diagnostic values.

## Limitations

In this study, we show a highly similar profile of cell antigens between APL and non-APL cases and highlight the uses of alternative cut-off points rather than a fixed 20% events for efficiently classify APL in the real-world. However, the sample size of the study is limited, while this is a single-center retrospective study. A further prospective study is required to confirm this finding, of which cell antigens as CD11b and CD35 should be collected adequately for the examination.

## Supplementary information

**Additional file 1: Figure S1.** Patient selection.

**Additional file 2: Table S1.** Clinical and laboratory characteristics.

**Additional file 3: Figure S2.** Flow cytometric plots of a case with APL (A) and a non-APL (B).

**Additional file 4: Figure S3.** Molecular and cytogenetic results of a case with APL (A) and a non-APL (B).

**Additional file 5: Table S2.** Antigen expression according to the cut-off value of 20% events.

**Additional file 6: Figure S4.** BMA analysis identified the five-factors optimal model for APL.

## Data Availability

The datasets generated during and/or analysed during the current study are available from the corresponding author on reasonable request.

## Abbreviations

APL: Acute promyelocytic leukemia
AML: Acute myeloid leukemia
ATRA: All-*trans* retinoic acid
ABL: Abelson
AUC: Area under the curve
BMA: Bayesian model averaging
CD: Cluster of differentiation
FISH: Fluorescence in situ hybridizations
HLA-DR: Human leukocyte antigen DR isotype
IREM2: Immune receptor expressed in monocytic derived cells
MPO: Myeloperoxidase
PML: Promyelocytic leukemia
RARA: Retinoic acid receptor alpha
RQ-PCR: Real-time quantitative polymerase chain reaction
RNA: Ribonucleic acid
ROC: Receiver operating characteristic
