# Supplementary figures and images for: The diagnostic power of CD117, CD13, CD56, CD64, and MPO in rapid screening acute promyelocytic leukemia
Source: BMC Res Notes. 2020 Aug 26;13:394. doi: 10.1186/s13104-020-05235-7 (PMC7449061; doi:10.1186/s13104-020-05235-7)

**Figure S1.** Patient selection

**
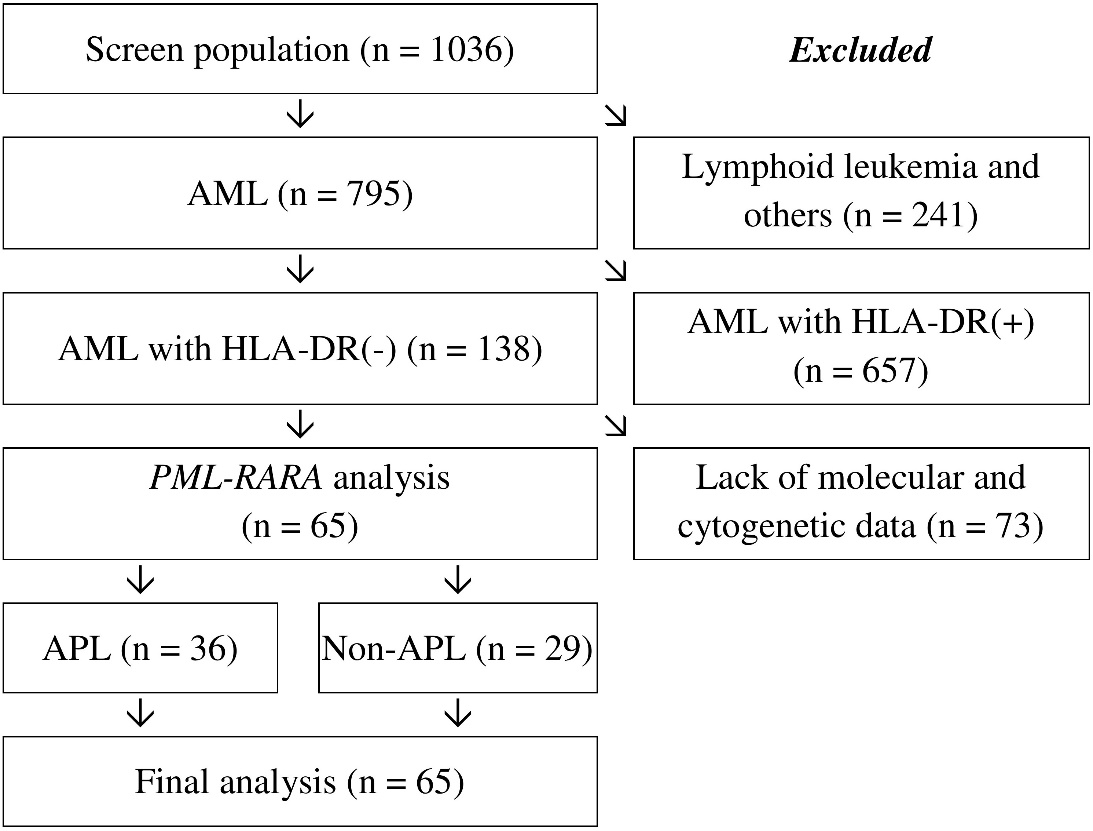
**

Supplement: Supplementary file 1 — Additional file 1: Figure S1. Patient selection. [file 13104_2020_5235_MOESM1_ESM.docx]

**Figure S2.** Flow cytometric plots of a case with APL (A) and a non-APL (B)

**
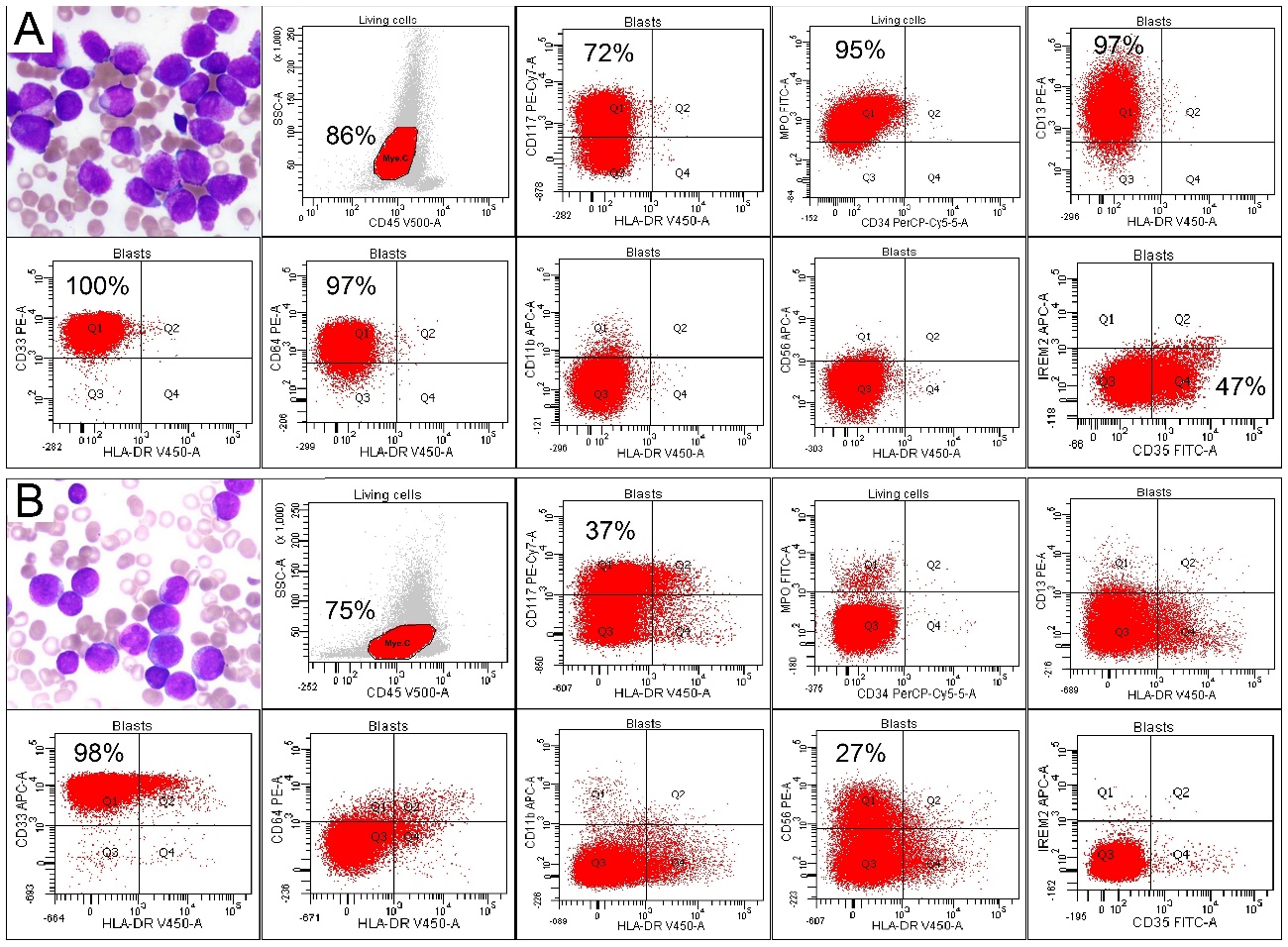
**

Supplement: Supplementary file 3 — Additional file 3: Figure S2. Flow cytometric plots of a case with APL (A) and a non-APL (B). [file 13104_2020_5235_MOESM3_ESM.docx]

**Figure S3.** Molecular and cytogenetic results of a case with APL (A) and a non-APL (B)

**
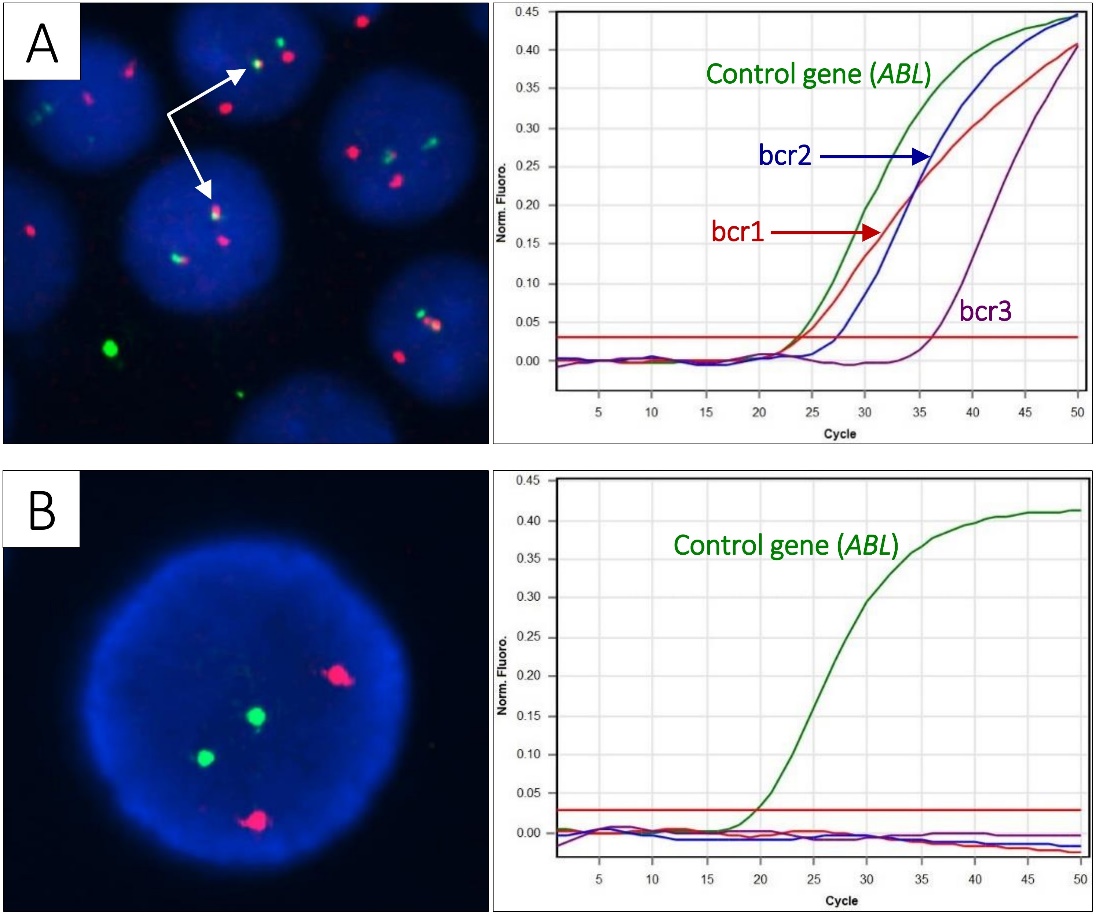
**

Supplement: Supplementary file 4 — Additional file 4: Figure S3. Molecular and cytogenetic results of a case with APL (A) and a non-APL (B). [file 13104_2020_5235_MOESM4_ESM.docx]

**Figure S4.** BMA analysis identified the five-factors optimal model for APL

**
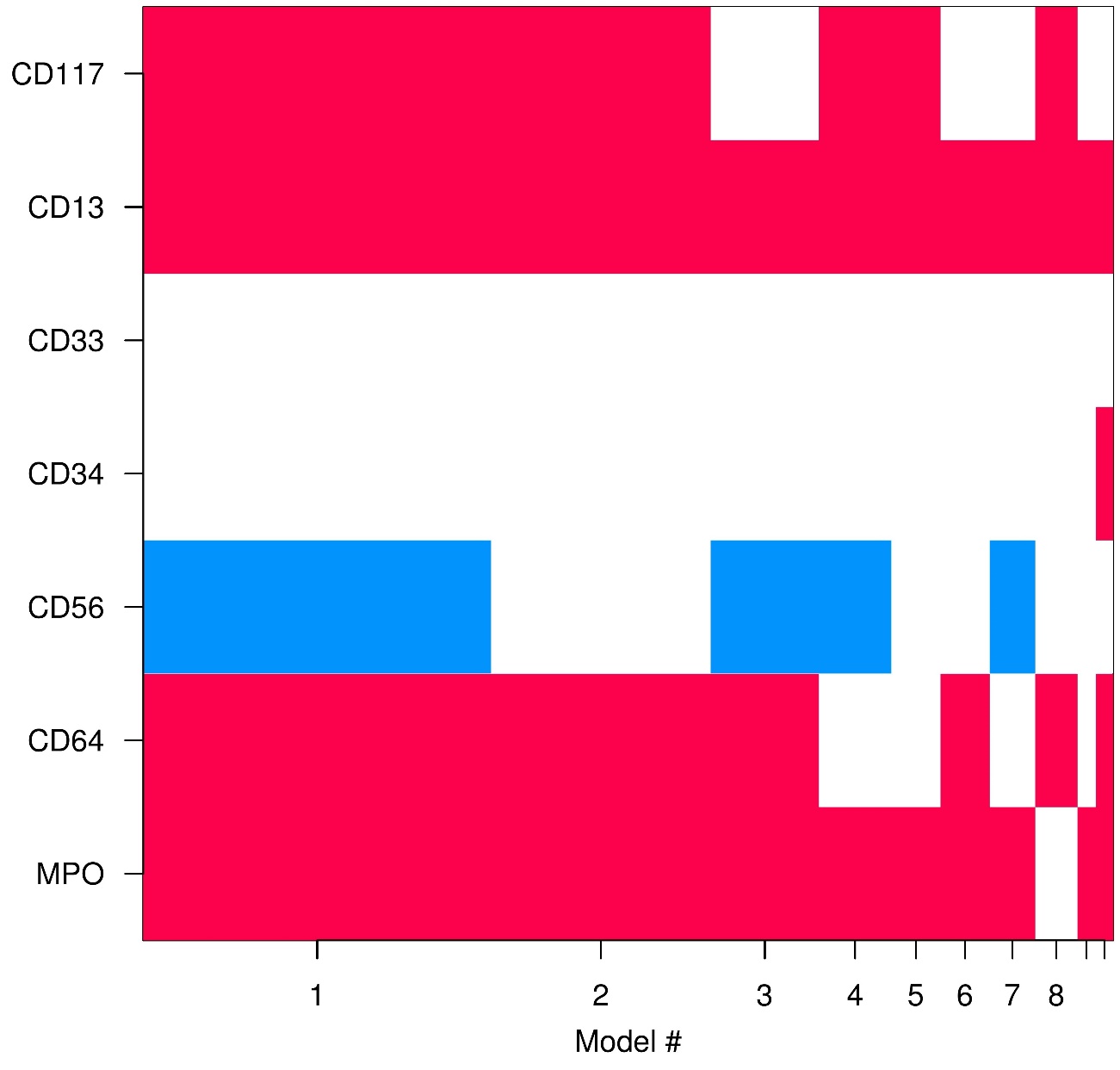
**

Supplement: Supplementary file 6 — Additional file 6: Figure S4. BMA analysis identified the five-factors optimal model for APL. [file 13104_2020_5235_MOESM6_ESM.docx]
