# Supplementary material for: The diagnostic power of CD117, CD13, CD56, CD64, and MPO in rapid screening acute promyelocytic leukemia
Source: BMC Res Notes. 2020 Aug 26;13:394. doi: 10.1186/s13104-020-05235-7 (PMC7449061; doi:10.1186/s13104-020-05235-7)
Supplement: Supplementary file 2 — Additional file 2: Table S1. Clinical and laboratory characteristics. [file 13104_2020_5235_MOESM2_ESM.docx]

**Table S1.** Clinical and laboratory characteristics

| **Variable** | **APL (n=36)** | **Non-APL (n=29)** |
| --- | --- | --- |
| Age, year (95%CI) | 46 (31–52) | 59 (53–64) |
| Gender (female/male) | 19/17 | 17/12 |
| Red blood cells, 10^12^/L (95%CI) | 2.58 (2.38–2.93) | 2.78 (2.24–3.04) |
| Hemoglobin, g/L (95%CI) | 80.0 (73.8–88.0) | 80.5 (67.3–91.7) |
| White blood cells, 10^9^/L (95%CI) | 6.7 (3.9–8.1) | 65.2 (34.5–106.8) |
| Platelets, 10^9^/L (95%CI) | 33.5 (29.6–43.7) | 38.5 (24.2–48.4) |
| t(15;17) translocation, % (95%CI) | 72.1 (37.0–84.3) | 0.0 (0.0–0.0) |
| *PML-RARA* transcripts, % (95%CI) | 82.9 (68.4–99.1) | 0.0 (0.0–0.0) |
