# Supplementary material for: The diagnostic power of CD117, CD13, CD56, CD64, and MPO in rapid screening acute promyelocytic leukemia
Source: BMC Res Notes. 2020 Aug 26;13:394. doi: 10.1186/s13104-020-05235-7 (PMC7449061; doi:10.1186/s13104-020-05235-7)
Supplement: Supplementary file 5 — Additional file 5: Table S2. Antigen expression according to the cut-off value of 20% events. [file 13104_2020_5235_MOESM5_ESM.docx]

**Table S2.** Antigen expression according to the cut-off value of 20% events

| **Variable** | **All (n=65)** | **APL (n=36)** | **Non-APL (n=29)** | ***P*-value** |
| --- | --- | --- | --- | --- |
| HLA-DR, +/- | 0/65 | 0/36 | 0/29 | - |
| CD117, +/- | 62/3 | 35/1 | 27/2 | 0.418 |
| CD34, +/- | 6/59 | 4/32 | 2/27 | 0.445 |
| CD11b*, +/- | 4/31 | 1/18 | 3/13 | 0.238 |
| CD13, +/- | 64/1 | 36/0 | 28/1 | 0.446 |
| CD14, +/- | 1/64 | 1/35 | 0/29 | 0.554 |
| CD33, +/- | 65/0 | 36/0 | 29/0 | - |
| CD35**, +/- | 14/22 | 13/7 | 1/15 | <0.001 |
| CD56, +/- | 14/51 | 2/34 | 12/17 | 0.001 |
| CD64, +/- | 47/18 | 33/3 | 14/15 | <0.001 |
| MPO, +/- | 62/3 | 36/0 | 26/3 | 0.084 |

*: data available in 35 cases; **: data available in 36 cases.
